# Evaluation of the Application of Italian National Guidelines for Prevention and Management of Dental Injuries in Developmental Age

**DOI:** 10.3390/ijerph17082875

**Published:** 2020-04-21

**Authors:** Maurizio Bossù, Francesco Covello, Gianni Di Giorgio, Stefania Zampogna, Valentina Talarico, Salvatore De Filippo, Antonella Polimeni, Stefano Di Carlo

**Affiliations:** 1 Department of Oral and Maxillofacial Sciences, “Sapienza” University of Rome, 00161 Rome, Italy; maurizio.bossu@uniroma1.it (M.B.); gianni.digiorgio@uniroma1.it (G.D.G.); antonella.polimeni@uniroma.it (A.P.); stefano.dicarlo@uniroma1.it (S.D.C.); 2 Pediatric First Aid, “Pugliese-Ciaccio” Hospital of Catanzaro, 88100 Catanzaro, Italy; stezampogna@gmail.com; 3 Department of Pediatrics, “Magna Graecia” University, “Pugliese-Ciaccio” Hospital of Catanzaro, 88100 Catanzaro, Italy; talaricovalentina@gmail.com; 4 Department of Oral Surgery, “Pugliese-Ciaccio” Hospital of Catanzaro, 88100 Catanzaro, Italy; sadefilippo@libero.it

**Keywords:** Trauma, tooth, fracture, luxation, first aid, knowledge

## Abstract

*Background*: The objective of this study is to evaluate the application of National guidelines for prevention and clinical management of traumatic dental injuries (NGPCMTDI) in developmental age published by the Italian Ministry of Health. *Methods*: In the present retrospective and multicenter study, 246 patients who underwent dental injury were selected to assess the management of the traumatic event compiled with the protocol provided by the National guidelines. Each health worker involved completed a form related to the dental injury in order to standardize the collected data. Two reference centers have been identified for data collection. Analyses for comparisons between groups were performed using the _X_^2^ test for categorical variables or by Fisher exact test as appropriate. Statistical significance was assumed at *p* < 0.05. *Results*: Evaluating the distribution by age we concluded that: 27.24% of the enrolled patients were aged 1–5 years, 51.63% 6–10 years, and 19.92% 11–17 years. The dental injuries occurred in 10.16% of the situations at home, 50.81% at school, 28.86% during recreation, and 9.35% at the gym. The deciduous dentition is involved in 34.96% of the traumas while the permanent dentition is involved in 69.51%. *Conclusion*: From the present study it emerged that the National guidelines are not uniformly applied.

## 1. Introduction

Italian National Guidelines for Prevention and Clinical Management of Traumatic Dental Injuries (TDIs) in developmental age, which in the present study we have taken as reference, indicate the strategies for prevention of traumatic dental injuries and health education, first aid protocol, certification of traumatic dental injuries, and identification of child abuse [1]. The primary goal of these guidelines is to delineate an approach for the immediate or urgent care of TDIs, to provide the best possible assistance [1].

TDIs occur with great frequency in preschool, school-age children, and young adults [2,3]. Guidelines for the evaluation and clinical management of TDIs have been developed by the International Association of Dental Traumatology [4,5,6], the American Academy of Pediatric Dentistry [7] and the American Association of Endodontists [8].

Management of injuries to the primary and permanent dentitions differs significantly, and separate guidelines have been developed in the overall management of trauma patients [7,8,9]. As emerges from scientific literature, only a few studies have assessed the experience necessary in the management of TDIs [10].

Trauma to the oral region accounts for 5% of all traumatic injuries [3,4,5,6,7,8,9,10,11,12]. Amongst the oral injuries, dental injuries are the most frequent, followed by soft-tissue injuries [13]. Dental injuries, in association with carious processes, are the pathologies most frequently observed in pediatric dentistry. This indicates that TDIs represent a global health problem. Studies in literature indicate that in industrialized countries, about one in five children had TDIs involving permanent teeth before leaving school [14]. The most exposed are children between the age of 1 and 2, regarding primary teeth, and the age of seven and nine, for permanent dentition [15]. Luxation injuries are the most common TDIs in the primary dentition, whereas crown fractures are more commonly reported for permanent dentition [2,16,17].

The early infancy, with the beginning of ambulation, with or without a walker, is the most frequent situation for dental injury due to the easy tendency to fall, resulting in collision with furniture or objects [18,19]. Obese children have greater exposure to falls; therefore, greater attention must be spent on the adoption of a suitable and correct nutrition lifestyle [20]. In children that present an increased overjet, possibly linked to the habit of sucking thumbs or pacifiers, there is a greater incidence of dental injuries [21]. It is important to resort to specialist dental visits in order to intercept and correct early dental protrusions [22]. In all these individuals as well as in particularly lively children, the use of mouthguards would be appropriate [23].

In managing a traumatic dental injury, the accuracy of the diagnosis, treatment plan and the regular check-ups over time are fundamental to ensure the success of the outcome. Guidelines should help the dentist, other health care professionals and whoever else may be involved in the treatment of dental injuries. The medical examination following the dental injury should be carried out as soon as possible, especially in the presence of the avulsion of permanent teeth [14].

The Italian National Guidelines for the prevention and clinical management of TDIs during developmental age indicate prevention and health education strategies, which derive from studies, for a total of 130 scientific works, based on primary and secondary prevention at home, school, during sport, and road collisions [1]. The implementation of suitable primary prevention measures, aiming to protect healthy subjects, depends on the correct information conveyed between pediatric dentists, dental hygienists, parents, schoolteachers, and sports teachers [1]. Secondary prevention measures are implemented when the damage has already occurred and are aimed at limiting the harmful effects through careful clinical evaluation and correct treatment of dental injury [1,24]. Finally, tertiary prevention of strict dental relevance, aims to reduce complications and restore the masticatory function [1]. The hypothesis of our study is to verify the implementation and efficiency of the application of the current national guidelines in order to meet the possible deficiencies. The aim of this study is to evaluate the adhesion and the modality of application of the National Guidelines for prevention and clinical management of TDIs in individuals in developmental age, published in November 2012 and updated in February 2018 by the Ministry of Health in Italy.

## 2. Materials and Methods

The study involved first aid physicians, pediatricians, hospital pediatricians, private dentistry professionals, universities, and hospitals. It is specified that the first aid physicians were assisted by dentists in the evaluation of the cases examined and that the pediatricians involved in the study have followed specific dental courses promoted by Italian scientific societies. Two reference centers have been identified: University of Rome “Sapienza” and “Pugliese-Ciaccio” Hospital of Catanzaro. Each center has identified the professional figures to be contacted, both within the public structure and on the territory. The pilot center was University of Rome “Sapienza”. The study started on December 1, 2014 with a patient enrollment time of about 12 months. Each health worker involved in the study was sent an evaluation form to be completed. This card was created specifically for the study in order to collect all data uniformly. For a form to be considered valid, it must be completed in its entirety. All the forms, completed correctly, were sent to the coordinating centers [Catanzaro-Rome] for data processing. Descriptive data for categorical variables are presented as percentages or ratios.

The parents or guardians of the patients who participated in the study signed an informed consent, included in the injury assessment form. The study was conducted in full accordance with the guidelines of the 1975 Declaration of Helsinki.

The inclusion criteria were as follows: Children with dental injuries from 1 to 17 years old, with signed informed consent by parents, complete and correct a compilation of the evaluation form together with the medical examination.

The exclusion criteria were as follows: Children who do not meet the inclusion criteria, patients unable to verbalize symptoms, incongruous compilation, or lacking the card. The only risk related to participation in this study is related to privacy. Every individual has been assigned with an alphanumeric code and to ensure privacy the data have been coded and stored in secure files. Every publication deriving from this research will guarantee the privacy of the child through the exclusion of personal data and other identification information.

A total of 246 patients (154 M, 92 F) were enrolled. All the demographic, clinical and anamnestic data were analyzed, as reported in the data collection form, on the modalities and characteristics of the traumatic event. In particular: place, dynamics, modality, symptoms, time of onset, and type of symptoms. In addition, they were analyzed by time of intervention on the TDIs, type of TDIs, predisposing factors, need for orthodontic treatment, wounds and lesions of the oral mucosa, sports activity at risk, and necessity to execute radiographies. Of the total: 61.79% of patients had a complete vaccination schedule.

Analyses for comparisons between groups were performed using the _X_^2^ test for categorical variables or by Fisher exact test as appropriate. Statistical significance was assumed at *p* < 0.05. Statistical analyses were performed using SPSS (version 18.0, SPSS Inc., Chicago, IL, USA).

## 3. Results

Evaluating the distribution by age we had:

1. 1–5 years 27.24% (32 F, 35 M),
2. 6–10 years 51.63% (43 F, 84 M),
3. 11–17 years 19, 92% (16 F, 33 M).

The TDIs occurred in 10.16% of situations at home (10 F, 15 M), 50.81% at school (47 F, 78 M), 28.86% during recreational activities (26 F, 45 M), and 9.35% at the gym (7 F, 16 M). In 77.64% of patients, the TDIs were consequent to falling, namely 113 males and 78 females (*p* = 0.04). In 45.93% of patients, the injury occurred during recreational activities, of which 10 were females and 32 males (*p* = 0.05). The TDIs were consequent to a direct impact in 94.31% (142 M, 90 F, *p* = 0.08) of the cases and to an indirect impact in 4.88% cases (10 M, 1 F, *p* = 0.06). The intervention time from the TDIs occurred in 2.44% of cases < 30 min, between 30–60 min in 3.66%, between 60–120 min in 24.39% and > 120 min in 30.49%. The symptoms reported by the patients were: pain in 41.87% of injuries, bleeding in 3.25%, headache in 1.63%; the time of symptoms onset is < 30 min in 12.60% of patients, between 30–60 min in 2.85%, between 60–120 min in 0.41% and > 120 min in 4.47%. The deciduous dentition was involved in 34.96% while the permanent dentition is 69.51%. The types of dental injury found were: fracture in 53.25% of patients, lateral luxation in 15.85%, avulsion in 13% (In 15.63% of the cases of avulsion there was adequate transport of the traumatized tooth), subluxation in 9.35%, concussion in 6.91%, and extrusive luxation in 5.28% (Table 1).

Overall, 6.50% of patients had an orthodontic therapy in place, 17.07% of patients presented malocclusion (13 F, 29 M) and 13.82% increased overjet (13 F, 21 M). The age of eruption of deciduous teeth was between 0–6 months in 39.84% and between 7–12 months in 23.58%. The age of eruption of the first permanent teeth was between 5–6 years in 30.89% of patients and between 6–8 years in 23.17%.

The 3.66% of patients had suffered from previous dental injury and 66.67% of those aged between 2–5 years, while 22.22% were aged between 6–10 years. At the physical examination, 95.12% of patients did not show changes in the state of consciousness and vital parameters.

A percentage of 28.86% of patients showed extraoral wounds: 43.66% on the upper lip, 11.27% on the lower lip, and 1.41% presented damage to the alveolar process. Of all the patients, 20.33% had lesions to the oral mucosa.

Local pain occurred in 43.90% of patients: spontaneously in 33.74%, to pressure in 9.76%, during mastication in 3.66%, in response to thermal stimulation in 6.91%, and in response to sweet foods in 0.41%.

Of the total patients, 14.23% of patients had lesions to the supporting structures of dental elements: In 10.16%, the gums has been involved; of these, 1.22% on the mandible and 4.47% on the maxilla. Correlations between genders and damage to supporting tissues and bone are shown in Table 2. A total of 80.08% of patients required Rx, and 14.63% required periodic orthodontic visits.

The association between age and predisposition showed that amongst patients presenting malocclusion, 26.9% are aged between 1 and 5 years (*p* = 0.88), 51.06% between 6 and 10 years (*p* = 0.69), and 21.9% of patients between 11–16 years (*p* = 0.43). Evaluating the increased overjet resulted in 25.4% of patients between 1–5 years (*p* = 0.47), 51.8% between the ages of 6–10 (*p* = 0.89), and 24.8% between 11–16 years (*p* = 0.33). The association between the dental visits and the pediatric visits shows that 88.07% of patients who go to the pediatrician do not undergo a dental visit (*p* = 0.03).

Further analysis shows how 5.28% of patients received information from leaflets at school and 44.72% of patients were given information from the pediatrician. About 21.14% of children practiced risky sports at the time of evaluation. The association between the dynamics and modalities of TDIs are shown in Table 3. The association between the location and means of dental injury shows that the most frequent modality was direct collision both at home (100% of the TDIs), at the gym (91.2%) and at school (100%). The association between the type of TDIs and place is shown in Table 4, between age and place of TDIs is shown in Table 5, between age and TDIs dynamics is highlighted in Table 6, between age and intervention time is shown in Table 7, and between age and type of TDIs is shown in Table 8. Finally, the association between age and extraoral wounds and oral mucosa lesions are shown in Table 9.

## 4. Discussion

The study showed that the majority of dental injuries occur in male patients (62.6%) compared to females (37.4%), scientific literature shows the same relation [25]. The percentages reported in literature are variable, but in accord to the study and inclusion criteria, the incidence of dental injury of male:female is 53.1% and 46.7% respectively [26,27]. The prevalence of TDIs in these premises, school and home, reflect the results of previous studies [18,23,28,29,30]. The school is the most frequent place and leads us to think that the recent recommended guidelines for prevention are not correctly applied. Regarding preventive measures in the school area, it would be desirable to organize meetings between the school staff and a specialized figure (dentist) in order to update all pertinent staff involving information on the national guidelines.

The location as well as other variables that referred to dental injuries, depend on the age of the child, establishing that for the youngest age group (1–2 years), the majority of TDIs are linked to falls and abuses. This latter hypothesis must always be considered in children under the age of 5 who have intra-oral lesions, injuries involving the lips, gums, tongue, palate, and serious lesions of the teeth [13,31].

The data obtained also show that as soon as children begin to gain confidence and coordination, the incidence of injuries decreases. This incidence increases again during the very active age group (8–12 years) [27] as a result of collisions, playgrounds and typical recreational activities linked to the age group until adolescence. In the age of adolescence, the most frequent injuries are referred to autonomous driving dynamics by cars and motorcycles [31]. 

According to the literature, the TDIs occurring by falling dynamics are 51.71%, followed by road accidents (22.90%), violence (5.67%), and sports (5.43%) [32].

The most frequent form of injury is direct collision (94.31%), whilst only a minority of TDIs are indirect (4.88%); the direct impact of a fall, based on our assessments, reached statistical significance (*p* < 0.003) in 79.74% of the study group. We observed that falling (79.74%) compared to playing (16%), often results in a direct impact. The male gender also presents TDIs (with statistical significance *p* < 0.04) mainly by falling. The TDIs have been observed above all in groups of patients presenting particular conditions: Obese children are more prone to falls, so further attention towards a more adequate diet becomes necessary also to avoid facial or dental injury [20]. Experimental works prior to our own have shown and elaborated that the main dynamics of TDIs are unintentional lesions such as collisions. In addition, children who are more exposed to risks tend to have more dental injuries, such as children subjected to bullying, hyperactive children, emotionally stressed children, and epileptic children (repeated falls). To these must be added children with cerebral palsy, difficulty in learning (due to lack of coordination), impaired hearing and vision, and children who make improper use of teeth and with oral piercings (especially amongst adolescents, due to chipping and fractures). Iatrogenic lesions (prolonged intubation) and sports injuries (low, medium and high risk sports) may cause TDIs [20].

The same study accurately distinguishes TDIs from intentional injury, such as TDIs from violence [20]. In our study, the TDIs occurred for 9.35% (22 subjects) at the gym. (7 F, 16 M). This result shows how in this premise there is not a complete adhesion to the national primary prevention guidelines for a safe practice of sport since only 2 subjects (10%) of the aforementioned 22 were wearing dental protection equipment. The most common symptom reported by our patients is pain (41.87%) followed by bleeding (3.25%), headache (1.63%) and later minor symptoms such as amnesia (only one case in our study) and difficulty in language.

Our study also analyzed the time of onset of the symptom of pain from the moment of TDI: in 12.60% of cases the pain appears after 30 min. from the TDI, between 30–60 min. in 2.85% of cases, between 60–120 min. in 0.41% of cases, after 120 min. in 4.47% of cases. The most affected teeth were permanent teeth (69.51%) compared to deciduous teeth (35%), in accordance with the literature. The study found that the most common type of dental injury is its fracture. In 53.25% of fracture cases, 90 patients were males and 41 females, with statistical significance with the male gender (*p* < 0.04). Following, there are lateral luxations in 15.85%, avulsion in 13.01% (in this category there was a significant correlation with female sex *p* < 0.02), and subsequently subluxation in 9.35%, concussion in 6.91%, and extrusive luxation in 5.28%. Observing the association between the type of TDI and the site, fractures were more frequent in the home environment (*p* < 0.03) in 93.2%, as was mobility in 65.21% (significance *p* < 0.007). In the school environment there was a greater association (*p* < 0.04) with concussions, which were registered in 74.4% of the patients. The present study also showed that the gums are involved in dental injury in 10.16% of cases. Analyzing the predisposing factors, we found that about 77.04% and 74.45% of the patients with TDI had malocclusion and increased overjet respectively. The forms of primary prevention reported in the national guidelines [1] indicate to intercept the aforementioned factors predisposing to dental trauma, and our study showed that in a high percentage of cases these forms of prevention were not implemented. Consequently, it would be desirable to organize, in schools, meetings between dentists, pupils and parents.

There was no different distribution of these predisposing factors in correlation with age. According to literature, traumatic lesions occur more often in patients with increased overjet and lip protrusion [27]. Children younger than 5 years, with an open front bite, have, as of probability, double the number of TDIs compared to their peers [20].

A large number of children, who suffered accidental damage to their maxillary incisors, had an increased overjet and insufficient resting lips. This could be due to the fact that the lips absorb the impact of materials colliding with the anterior teeth, minimizing the possibility of a fracture; moreover, an increased overjet means a greater exposure of the teeth, which predisposes to dental injuries [27].

The most frequently observed post-traumatic injuries were extraoral wounds, the latter being more common in children between the ages of 6 and 10 (*p* = 0.01) and 11–16 years (*p* = 0.004). Analyzing the lesions of the oral mucosa, they were distributed more frequently in children aged between 1–5 years (*p* = 0.002) and 6–10 years (*p* = 0.006).

As it is clearly shown in the guidelines [1], the crucial element in the correct management of TDIs is the time elapsed between the event and a possible therapeutic approach. In the presence of certain types of dental injury [permanent tooth avulsion], the time between the trauma and the re-implanting, that should be before 2 h from trauma, can significantly compromise the final prognosis [1,33]. When the avulsed teeth are re-implanted after a maximum of 5 min, 73% will have a normal healing prognosis, a percentage that drops to 50% if they spend more than 10 min outside the alveolar socket [31].

More than 30% of the children observed in our study were taken to a medical examination after 2 h from the injury; only in younger children, under the age of 5, there has been a tendency to take the child to a medical examination within 120 min (*p* = 0.01). The intervention time from the TDIs occurred in 2.44% of cases < 30 min, between 30–60 min in 3.66%, between 60–120 min in 24.39%, and > 120 min in 30.49%. This result shows how in a high number of cases the national guidelines were not correctly applied.

Regarding the diagnostic approach, the study shows that a percentage of 81% of patients performed an intraoral Rx examination after TDI; this result is in accordance with the recommendations of the current guidelines [1]. The most suitable radiographic examination is the intraoral Rx. In the presence of penetrating wounds of the lips, the radiography of the soft parts may be necessary for the identification of foreign bodies.

A percentage of 14.63% of the children in the study were subjected to orthodontic therapy, according to the current guidelines [1], especially in the presence of avulsion of a permanent tooth, dental dislocation, crown fracture, and root with or without pulp exposure.

The management of injuries to the deciduous teeth provides for the prevention of damage to permanent teeth, especially following dislocation of the anterior incisors [33].

Deciduous avulsed teeth should not be replanted because they could be a cause for damage to the developing dental germ [1,33]; however, the tooth should be examined to make sure that the entire crown and root are present. The fracture on the deciduous tooth, although rare, requires immediate action to prevent infections and inflammation [33].

Knowing how to correctly handle dental injury by any medical figure or family pediatrician is a fundamental necessity, given the high incidence of cases in the common clinical practice. The application of simple and clear maneuvers significantly modifies the final prognosis of TDIs. A deficiency found in our study was the one related to the collaboration between pediatricians and dentists.

TDIs are a significant problem in young people and are followed by incidence of dental caries and periodontal disease. Although dental injuries occur at any age, they are more frequent between 2 and 5 years. Males continue to be more exposed, probably due to behavioral factors and greater hyperactivity.

Information, control and rehabilitation actions should be amplified in regions with higher incidence and higher density of TDIs, thus providing a greater degree of precision and effectiveness in the management of such emergencies and improving health care.

During a fall, the prevailing modality of impact is the direct one. The medium and long-term consequences of TDI affecting a deciduous tooth is the most serious complication to consider given the possibility that the gem of permanent dentition will be compromised. A TDI on a permanent tooth can induce complications that can go from ankylosis to re-impaction, up to the involvement of the surrounding bone structure with loss of the tooth and the alveolar bone.

Most of the available literature points out how treatment of TDIs is unsatisfactory. It is recognized that the prognosis of TDIs depends on the time between the diagnosis and the start of treatment.

Emergency dental treatment by a doctor is necessary if the dentist is not available. Often the first figure managing a TD is the first aid doctor [14].

With the term of first aid of dental traumas in pediatric age we want to indicate all those procedures that can be applied by the figure who is facing this event, to significantly modify the prognosis [33].

In these circumstances, the knowledge of a well-coded procedure to manage the traumatic event allows a more adequate and correct resolution of the problem [31].

In this regard, the pediatrician, more than any other medical figure, should have the necessary knowledge to correctly handle emergencies such as these, as well as being able to provide parents with professional advice for all aspects concerning the health of the child. This should include basic knowledge of the most common injuries [15]. The limit of our study is represented by not having been able to include minor dental traumas, compared to those reported by us, which could however lead to the onset of complications affecting the elements involved. Our suggestion for the future is to introduce interdisciplinary training events between dentists and the different medical figures who deal with patients in the evolutionary age.

## 5. Conclusions

The results of this study have shown that the National guidelines for the prevention and clinical management of TDIs in developmental age are not uniformly applied. How it is possible to understand from our results, if the correct primary, secondary and tertiary prevention strategies are correctly applied, surely decreases the number of patients with dental trauma and the clinical management of the latter will certainly be better

In order to identify the most suitable type of clinical approach, based on the type of TDI and the affected dental element, precise indications are found in the literature.

## Figures and Tables

**Table 1:** Type of dental injury.

Type of Dental Injury	Total (TOT)	%	Male (M)	%	Female (F)	%	*p*
Avulsion	32	13.01	14	43.7	18	56.25	*p* = 0.02
Extrusive luxation	13	5.28	9	69.23	4	30.7	*p* = 0.77
Fracture	131	53.25	90	68.7	41	31.3	*p* = 0.04
Concussion	17	6.91	12	70.6	5	29.4	*p* = 0.66
Lateral luxation	39	15.85	24	61.5	15	38.5	*p* = 0.99
Subluxation	23	9.35	12	52.17	11	48	*p* = 0.36

**Table 2:** Association between gender and traumatic injuries to supporting tissues and bone structures.

Supporting Tissues and Bone Structures	TOT	%	M	%	F	%	*p*
Gum	25	10.16	10	40	15	60	*p* = 0.001
Jaw	3	1.22	2	66.6	1	33.33	*p* = 0.99
Maxilla	11	4.47	5	44.54	6	54.54	*p* = 0.33

**Table 3:** Association between type of injury and dynamics.

Type of Injury	Fall	*p*	Playing	*p*
Direct impact	79.74	0.003	16%	0.06
Indirect impact	54.54	0.07	36.36%	0.09

**Table 4:** Association between type of injury and place of the event.

Type of Injury	Home	*p*	School	*p*	Gym	*p*
Avulsion	18.7%	*p* = 0.11	34.3%	*p* = 0.06	3.1%	*p* = 0.32
Lateral luxation	23%	*p* = 0.13	46%	*p* = 0.78	15.3%	*p* = 0.34
Extrusive luxation	5%	*p* = 0.38	59%	*p* = 0.29	10%	*p* = 0.76
Fracture	6%	*p* = 0.03	49.6%	*p* = 0.70	11.5%	*p* = 0.27
Concussion	0%	*p* = 0.23	76.4%	*p* = 0.04	11.7%	*p* = 0.66
Subluxation	34.7%	*p* = 0.007	39.1%	*p* = 0.27	0%	*p* = 0.14

**Table 5:** Association between age and event place.

Age	Home	*p*	School	*p*	Gym	*p*
1–5 years	64%	*p* = 0.001	31%	*p* = 0.25	4.54%	*p* = 0.01
6–10 years	34.7%	*p* = 0.03	57.7%	*p* = 0.09	45.45%	*p* = 0.51
11–16 years	4%	*p* = 0.03	11.38%	*p* = 0.007	50%	*p* = 0.001

**Table 6:** Association between age and event dynamics.

Age	Fall	*p*	Playing	*p*
1–5 years	27.51%	*p* = 0.99	24.39%	*p* = 0.70
6–10 years	59%	*p* = 0.01	39.02%	*p* = 0.08
11–16 years	84%	*p* = 0.003	63.4%	*p* = 0.009

**Table 7:** Association between age and time from the injury.

Age	<30 min	*p*	30–60 min	*p*	60–120 min	*p*	>120 min	*p*
1–5 years	66.66%	*p* = 0.04	22.22%	*p* = 0.99	14.03%	*p* = 0.01	31%	*p* = 0.06
6–10 years	16.66%	*p* = 0.11	55.5%	*p* = 0.99	61.4%	*p* = 0.98	50.29%	*p* = 0.58
11–16 years	16.66%	*p* = 0.99	22.22%	*p* = 0.99	24.56%	*p* = 0.34	18.7%	*p* = 0.49

**Table 8:** Association between age and type of dental injury.

Age	Avulsion	*p*	Dislocation	*p*	Concussion	*p*	Luxation	*p*	Mobility	*p*
1–5 years	45.16%	*p* = 0.02	69.23%	*p* = 0.0016	43.75%	*p* = 0.0015	42.10%	*p* = 0.04	48%	*p* = 0.02
6–10 years	35.48%	*p* = 0.05	23.07%	*p* = 0.04	50%	*p* = 0.99	39.47%	*p* = 0.11	39.13%	*p* = 0.19
11–16 years	19.35%	*p* = 0.99	7.69%	*p* = 0.14	6.25%	*p* = 0.25	18.42%	*p* = 0.99	20.90%	*p* = 0.58

**Table 9:** Association between age and extraoral wounds/lesions of the oral mucosa.

Age	Extraoral Wounds	*p*	Lesions of the Oral Mucosa	*p*
1–5 years	27%	*p* = 0.87	49%	*p* = 0.002
6–10 years	40%	*p* = 0.01	34.7%	*p* = 0.006
11–16 years	33%	*p* = 0.004	16.3%	*p* = 0.55

## References

[1] (2018). National Guidelines for Prevention and Clinical Management of Traumatic Dental Injuries in Developmental Age. http://www.salute.gov.it/imgs/C_17_pubblicazioni_2755_allegato.pdf.

[2] Andreasen J.O., Andreasen F.M., Andersson L. (2007). Textbook and Color Atlas of Traumatic Injuries to the Teeth.

[3] Petersson E.E., Andersson L., Sorensen S. (1997). Traumatic oral vs non-oral injuries. Swed. Dent. J..

[4] Flores M.T., Andersson L., Andreasen J.O., Bakland L.K., Malmgren B., Barnett F., Bourguignon C., Di Angelis A., Hicks L., Sigurdsson A. (2007). Guidelines for the management of traumatic dental injuries. 1. Fractures and luxations of permanent teeth. Dent. Traumatol..

[5] Flores M.T., Andersson L., Andreasen J.O., Bakland L.K., Malmgren B., Barnett F., Bourguignon C., Di Angelis A., Hicks L., Sigurdsson A. (2007). Guidelines for the management of traumatic dental injuries. 11. Avulsion of permanent teeth. Dent. Traumatol..

[6] Flores M.T., Malmgren B., Andersson L., Andreasen J.O., Bakland L.K., Barnett F., Bourguignon C., Di Angelis A., Hicks L., Sigurdsson A. (2007). Guidelines for the management of traumatic dental injuries. 111. Primary Teeth. Dent. Traumatol..

[7] American Academy of Pediatric Dentistry (2001). Guideline on Management of Acute Dental Trauma. Council of Affairs. Reference Manual 2002–2003.

[8] American Association of Endodontists (2004). Recommended Guidelines of the American Association of Endodontists for the Treatment of Traumatic Dental Injuries.

[9] Australian Dental Association (ADA) (2005). Community Oral Health Promotion, Prevention & Management of Oral Injuries.

[10] Jackson N.G., Waterhouse P.J., Maguire A. (2005). Management of dental trauma in primary care: A postal survey of general dental practitioners. Br. Dent. J..

[11] Glendor U., Andersson L., Pine C., Harris R. (2007). Public health aspects of oral diseases and disorders; dental trauma. Community Oral Health.

[12] Glendor U., Halling A., Andersson L., Eilert-Petersson E. (1996). Incidence of traumatic tooth injuries in children and adolescents in the county of Vastmanland, Sweden. Swed. Dent. J..

[13] Malmgren B., Andreasen J.O., Flores M.T., Robertson A., DiAngelis A.J., Andersson L., Cavalleri G., Cohenca N., Day P., Hicks M.L. (2012). International Association of Dental Traumatology. International Association of Dental Traumatology guidelines for the management of traumatic dental injuries: 3. Injuries in the primary dentition. Dent. Traumatol..

[14] Emerich K., Gazda E. (2010). Review of recommendations for the management of dental trauma presented in first-aid textbooks and manuals. Dent. Traumatol..

[15] Baginska J., Wilczynska-Borawska M. (2012). First-aid algorithms in dental avulsion. J. Sch. Nurs..

[16] Flores M.T. (2002). Traumatic injuries in the primary dentition. Dent. Traumatol..

[17] Kramer P.F., Zembruski C., Ferreira S.H., Feldens C.A. (2003). Traumatic dental injuries in Brazilian preschool children. Dent. Traumatol..

[18] De Lourdes Drachler M., de Carvalho Leite J.C., Marshall T., Hess Almaleh C.M.A., Feldens C.A., Vitolo M.R. (2007). Effects of the home environment on unintentional domestic injuries and related health care attendance in infants. Acta Pediatrica.

[19] Noori A.J., Al-Obaidi W.A. (2009). Traumatic dental injuries among primary school children in Sulaimani city, Iraq. Dent. Traumatol..

[20] Glendor U. (2009). Aetiology and risk factors related to traumatic dental injuries—A review of the literature. Dent. Traumatol..

[21] Gupta S., Kumar-Jindal S., Bansal M., Singla A. (2011). Prevalence of traumatic dental injuries and role of incisal overjet and inadequate lip coverage as risk factors among 4–15 years old government school children in Baddi-Barotiwala Area, Himachal Pradesh, India. Med. Oral Patol. Oral Cir. Bucal.

[22] Ankola A.V., Hebal M., Sharma R., Nayak S.S. (2013). Traumatic dental injuries in primary school children of South India—Report from district-wide oral health survey. Dent. Traumatol..

[23] Spinas E., Savasta A. (2007). Prevention of traumatic dental lesions: Cognitive research on the role of mouth guard during sport activities in paediatric age. Eur. J. Paediatr. Dent..

[24] Levin L., Zadik Y. (2012). Education on and prevention of dental trauma: It’s time to act!. Dent. Traumatol..

[25] Dua R., Sharma S. (2012). Prevalence, causes, and correlates of traumatic dental injuries among seven-to-twelve-year-old school children in Dera Bassi. Contemp. Clin. Dent..

[26] Carvalho M.L., Moysés S.J., Bueno R.E., Shimakura S., Moysés S.T. (2010). A geographical population analysis of dental trauma in schoolchildren aged 12 and 15 in the city of Cuturiba-Brazil. BMC Health Serv. Res..

[27] Govindarajan M., Reddy V.N., Ramalingam K., Durai K.S., Rao P.A., Prabhu A. (2012). Prevalence of traumatic dental injuries to the anterior teeth among three to thirteen years old school children of Tamilnadu. Contemp. Clin. Dent..

[28] Chan Y.M., Williams S., Davidson L.E., Drummond B.K. (2011). Orofacial and dental trauma of young children in Dunedin, New Zealand. Dent. Traumatol..

[29] Al-Obaida M. (2010). Knoeledge and management of traumatic dental injuries in a group of Saudi primary school’s teachers. Dent. Traumatol..

[30] La Monaca G., Pranno N., Vozza I., Annibali S., Polimeni A., Bossù M., Cristalli M.P. (2019). Sequelae in permanent teeth after traumatic injuries to primary dentition. Minerva Stomatol..

[31] Emerich K., Wyszkowski J. (2010). Clinical practice: Dental trauma. Eur. J. Pediatr..

[32] Guedes O.A., Alencar AH G.D., Lopes L.G., Pécora J.D., Estrela C. (2010). A retrospective study of traumatic dental injuries in a Brazilian dental urgency service. Braz. Dent. J..

[33] Brullmann D., Schulze R.K., D’Hoedt B. (2010). The treatment of anterior dental trauma. Dtsch. Ärzteblatt Int..

